# HEMD: An Integrated Tool of Human Epigenetic Enzymes and Chemical Modulators for Therapeutics

**DOI:** 10.1371/journal.pone.0039917

**Published:** 2012-06-25

**Authors:** Zhimin Huang, Haiming Jiang, Xinyi Liu, Yingyi Chen, Jiemin Wong, Qi Wang, Wenkang Huang, Ting Shi, Jian Zhang

**Affiliations:** 1 Department of Pathophysiology and Key Laboratory of Cell Differentiation and Apoptosis of Chinese Ministry of Education, School of Medicine, Shanghai Jiao-Tong University, Shanghai, China; 2 Institute of Biomedical Sciences and School of Life Sciences, East China Normal University, Shanghai, China; 3 Shanghai Key Laboratory of Tumor Microenvironment and Inflammation, Shanghai Jiao-Tong University, Shanghai, China; Bellvitge Biomedical Research Institute (IDIBELL), Spain

## Abstract

**Background:**

Epigenetic mechanisms mainly include DNA methylation, post-translational modifications of histones, chromatin remodeling and non-coding RNAs. All of these processes are mediated and controlled by enzymes. Abnormalities of the enzymes are involved in a variety of complex human diseases. Recently, potent natural or synthetic chemicals are utilized to establish the quantitative contributions of epigenetic regulation through the enzymes and provide novel insight for developing new therapeutics. However, the development of more specific and effective epigenetic therapeutics requires a more complete understanding of the chemical epigenomic landscape.

**Description:**

Here, we present a human epigenetic enzyme and modulator database (HEMD), the database which provides a central resource for the display, search, and analysis of the structure, function, and related annotation for human epigenetic enzymes and chemical modulators focused on epigenetic therapeutics. Currently, HEMD contains 269 epigenetic enzymes and 4377 modulators in three categories (activators, inhibitors, and regulators). Enzymes are annotated with detailed description of epigenetic mechanisms, catalytic processes, and related diseases, and chemical modulators with binding sites, pharmacological effect, and therapeutic uses. Integrating the information of epigenetic enzymes in HEMD should allow for the prediction of conserved features for proteins and could potentially classify them as ideal targets for experimental validation. In addition, modulators curated in HEMD can be used to investigate potent epigenetic targets for the query compound and also help chemists to implement structural modifications for the design of novel epigenetic drugs.

**Conclusions:**

HEMD could be a platform and a starting point for biologists and medicinal chemists for furthering research on epigenetic therapeutics. HEMD is freely available at http://mdl.shsmu.edu.cn/HEMD/.

## Introduction

Epigenetics is the study of any potentially stable and heritable change in gene expression or cellular phenotype that occurs without changes in DNA [1], [2]. Epigenetic regulation of gene expression can significantly alter the cellular phenotype due to their ability to activate/silence genes and is mediated through chromatin composed of DNA, histones, non-histone proteins, and non-coding RNA [3]. Currently, there are at least four types of epigenetic regulation: DNA methylation, post-translational modifications of histones (from relatively small groups such as methyl, acetyl, and phosphoryl groups to the attachment of larger moieties such as poly (ADP-ribose) and small ubiquitin-like modifier (SUMO)), ATP-dependent chromatin remodeling (eviction, deposition, or sliding of nucleosomes along DNA), and non-coding RNA regulation (microRNA, small interfering RNA, piwi-interacting RNA, etc) [2], [4], [5]. These modifications are mediated and controlled by a group of enzymes, which define as epigenetic enzymes by Copeland *et al* [6].

Epigenetic enzyme-mediated control of gene transcription is a critical aspect of embryonic development and continues to play a role in gene regulation and genome stability throughout the lifespan of an organism [7]. The mechanism is often dysregulated with aberrant gene expression and repression in human diseases (e.g. cancer, depression, diabetes mellitus, and inflammatory disease) and the abnormalities have been found to be associated with amplification, mutation, and other alterations of epigenetic enzymes [8], [9], indicating that specific classes of diseases might benefit from epigenetic-targeting therapies. Therefore, identifying the most appropriate enzymes that should be targeted in the cases of different diseases is a fundamental prerequisite for epigenetic therapeutics.

In recent years, remarkable progress has been made in target identification, drug discovery, and clinical validation for epigenetic therapeutics [10]. Three classes of epigenetic enzymes have been successfully targeted by small chemical modulators that have reached clinical trials for specific therapeutics: the DNA methyltransferases (e.g., Azacitidine, Decitabine), histone deacetylases (e.g., Vorinostat, Romidepsin), and Aurora-B kinases (e.g., Tozasertib, Danusertib) [6]. Some epigenetic enzyme classes have been demonstrated to have strong disease association and are currently being targeted by small molecular modulators in preclinical discovery programs at a number of academic, industry, and government laboratories [11]–[14]. With substantial enthusiasm for the development and implementation of epigenetic therapies, more and more modulators emerged one after another for orphan epigenetic enzymes; on the other hand, known epigenetic modulators may not just work as single agents but rather as components of combination therapies. Therefore, a complete understanding of the chemical epigenomic landscape for chemical-enzyme pairs is necessary for the commencement of more specific and effective therapeutic strategies.

Nowadays, most of the existing resources devoted to epigenetics focus mainly on detailed information about epigenetic features in methylated DNAs [15], [16], modified chromatin proteins [17]–[19], and associated phenotypes [20]–[22] but less on their therapeutics―epigenetic enzymes and modulators. Specialized databases and analysis systems dedicated to epigenetic enzymes and modulators are becoming crucial for a better understanding epigenetic mechanisms of enzymes and designing modulators for therapeutics. To fill this gap, we have developed HEMD, an integrated database of human epigenetic enzymes and their modulators focused on epigenetic therapeutics. This is the first online database, to our knowledge, that focuses on exhaustive information from epigenetic regulation, related disorders to therapeutics in the context of relationship between 269 human enzymes and 4377 modulators, together with their statistical evaluation, references to the scientific literature, and cross-links to other associated databases, such as Enzyme Nomenclature [23], NCBI Epigenomics [15], DAnCER [20], GenBank [24], Uniprot [25], etc. Furthermore, BLAST (basic local alignment search tool) search engine for enzymes and a chemical structure search engine for small modulators are available as web-based tools for epigenetic molecule recognition. Taken together, HEMD is a comprehensive resource that could provide useful information and tool for the investigation of epigenetic mechanisms and novel drug design.

## Materials and Methods

First, abstracts of PubMed were automatically filtered for relevant articles in the “DNA methylation”, “histones modification”, “chromatin remodeling”, and “non-coding RNA”. The names of proteins were then extracted from the abstracts to clusters by a protein name dictionary constructed from UniProt [25], retrieving ∼400 distinct biological proteins in human. A team of scientists manually processed the papers with respect to the clustered names. With at least three cases of experimental evidence in biochemistry, crystal structure complex and domain analysis, 269 proteins supporting their functional regulation of gene expression by epigenetic manner, were verified as epigenetic enzymes for deposition into the HEMD. All proteins in the HEMD were annotated with gene information, biological function, natural mutations, and related diseases extracted from GenBank [24], Uniprot [25], Enzyme Nomenclature [23] and original literature. An up-to-date synchronization on available structures of epigenetic enzymes from PDB [26] is present and their structural classification SCOP [27] and CATH [28] based on the PDB ID are also labeled. Theoretical models of epigenetic enzymes without structures were generated with I-TASSER [29] or built manually using Modeller [30] when C-score of the best I-TASSER model is below −1.5 or high-homologous oligomeric templates are available in PDB. All structures are downloadable as PDB files. Notably, extensive descriptions of binding sites on both substrates and modulators were separately summarized from literature and the sites were always highlighted in the diagram of protein topologies [31], [32] if they have been explicitly validated by biochemistry or structural biology.

Second, after all epigenetic enzymes with relevant annotation information were collected, we further searched for epigenetic modulators for the 269 epigenetic enzymes. All the abstracts from PubMed, United States Patent and European Patent files containing “modulator/effector/activator/inhibitor/agonist/antagonist” in combination with the name of the collected epigenetic enzymes were curated and then manually identified as the final set, resulting in the collection of 4377 chemical epigenetic modulators with respective references. Meanwhile, publicly available binding affinities and test methods of the epigenetic modulators to their epigenetic targets were also obtained from the references. Among the epigenetic modulators in the HEMD, those that increase a particular protein function, for example, catalytic rate, are classified as “epigenetic activator” or “A”. Those that decrease a particular protein function are classified as “epigenetic inhibitor” or “I”. The remaining modulators, which have dual effects on activation and inhibition in a concentration-dependent manner, are classified to the “epigenetic regulator” or “R” category. Since epigenetic modulators were initially identified from endogenous ligands and then widely accepted for the development of novel types of drugs, the tags “Endogenous” and “Druggable” in the HEMD differentiate epigenetic modulators produced *in vivo* and designed for drug use, respectively. In addition, important physicochemical properties used in drug discovery, such as logP, PSA, the number of rotatable bonds, etc., were calculated on the epigenetic modulators by Filter Program from Openeye (http://www.eyesopen.com). Each modulator in the HEMD is downloadable as 2D mol and 3D mol2 files.

## Results

### The HEMD database

**Figure 1:**
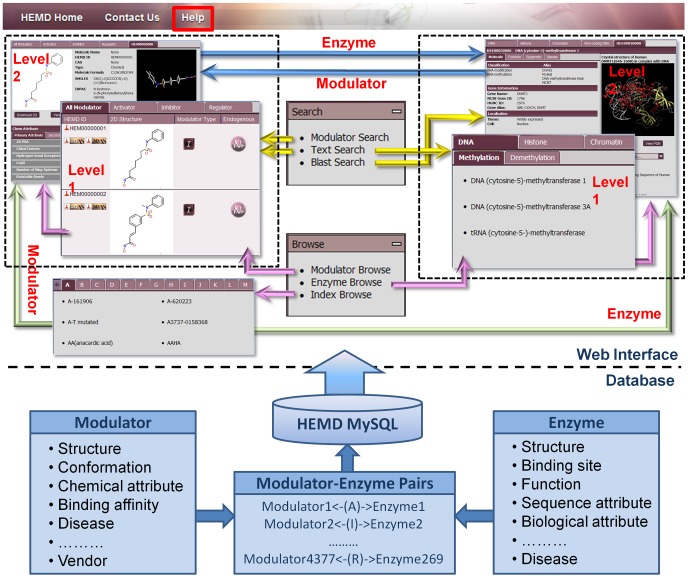
Web interface and back-end database in HEMD. Key interface screenshots showing the interrelation of tools and user can directly view from “Browse” menu or start search by “Search” menu. All recorders are deposited in MySQL and some kind of important data for epigenetic therapeutics has been summarized in the lower part of the diagram.

HEMD is an integrated repository on epigenetic enzymes and chemical modulators for therapeutics, which was manually curated from original literature. In total, 269 epigenetic enzymes and 4377 chemical modulators identified from in vitro binding to the epigenetic enzymes were deposited and fully annotated by the database developers and experts in the field (Figure 1).

**Figure 2:**
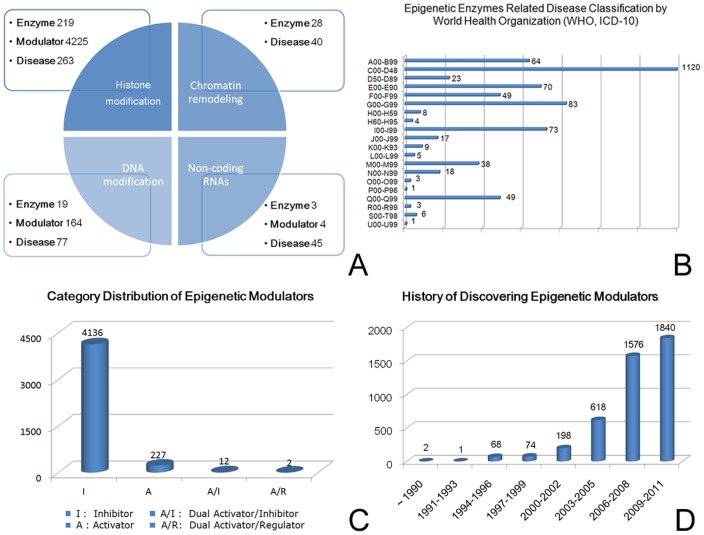
Statistics on the epigenetic enzymes and modulators. (A) Class distribution of epigenetic enzymes. (B) The classification of epigenetic enzymes related disease by WHO codes. A00–B99: Certain infectious and parasitic diseases, C00–D48: Neoplasms, D50–D89: Diseases of the blood and blood-forming organs and certain disorders involving the immune mechanism, E00–E90: Endocrine, nutritional and metabolic diseases, F00–F99: Mental and behavioural disorders, G00–G99: Diseases of the nervous system, H00–H59: Diseases of the eye and adnexa, H60–H95: Diseases of the ear and mastoid process, I00–I99: Diseases of the circulatory system, J00–J99: Diseases of the respiratory system, K00–K93: Diseases of the digestive system, L00–L99: Diseases of the skin and subcutaneous tissue, M00–M99: Diseases of the musculoskeletal system and connective tissue, N00–N99: Diseases of the genitourinary system, O00–O99: Pregnancy, childbirth and the puerperium, P00–P96: Certain conditions originating in the perinatal period, Q00–Q99: Congenital malformations, deformations and chromosomal abnormalities, R00–R99: Symptoms, signs and abnormal clinical and laboratory findings, not elsewhere classified, S00–T98: Injury, poisoning and certain other consequences of external causes, U00–U99: Codes for special purposes. (C) Category distribution of epigenetic modulators. (D) History of discovering epigenetic modulators.

Epigenetic enzymes in HEMD cover four parts, of which 19 of the epigenetic proteins are from DNA methylation, 219 from histone modification, 28 from chromatin remodeling and 3 from non-coding RNA (Figure 2). Crystal structures of 929 redundant proteins were extracted from PDB and 142 epigenetic enzymes have been resolved. Based on the known structures, theoretical 3D models of the remaining 127 enzymes without crystal structures are constructed and downloadable from HEMD. Ninety-two modulator binding sites of the enzymes were identified from crystal complexes. By analyzing the occurrence of structural domains in the epigenetic enzymes represented in Pfam [31], two kinds of known domains, DNA/RNA/Histone binding domain and catalytic domain, which are the structural basis for epigenetic function are found in 43% and 74% of the 269 epigenetic enzymes, respectively. In addition, success in developing epigenetic therapeutics relies heavily on identifying the most relevant diseases to target, therefore 317 diseases from abnormal epigenetic enzymes have been carefully referenced and exhaustively described in HEMD, including 113 types of cancer and 204 non-oncology disorders.

Among 4377 epigenetic chemical modulators in current HEMD, 241 activators, 4148 inhibitors, and 2 regulators were revealed to bind to corresponding epigenetic enzymes, and only 14 modulators (0.32%) have multiple effects on different epigenetic systems (Figure 2), finally resulting in 7016 epigenetic interactions between the enzymes and chemical modulators in HEMD. The chemical modulators range from organic small molecules (4053, 92.60%), peptides (249, 5.69%), nucleotides (61, 1.39%), to salts (14, 0.32%). Since almost all epigenetic hits/leads/drugs were initially derived from endogenous molecules and then screened and modified in drug discovery, HEMD now holds 176 endogenous epigenetic seeds and 4199 compounds in the pipeline of drug discovery.

### Web interface

HEMD provides a variety of interfaces and graphical visualizations to facilitate viewing and analysis of the epigenetic molecules (enzymes and modulators) from structures, functions and related therapeutics. As shown in Figure 1, HEMD presents the three browsing starting points and three search options. To visually understand the data in HEMD, browsing and searching tools are fully crosslinked. One can quickly jump from search results to their full information pages so that the users can analyze data more efficiently. For example, users can start by searching the name of an epigenetic molecule and visualize a complete description on the information page and then download the specific molecule for further review.

HEMD supports ﬂexible query for various epigenetic molecules and related function and therapeutic annotation by providing three “Search” tools–“Blast search”, “Modulator search” and “Text search”. “Blast search” is powered by BLASTp [33] and is particularly useful as it allows users to quickly identify epigenetic enzyme by comparing the query proteins to known epigenetic enzymes in HEMD. The search is triggered by pasting a FASTA format sequence and pressing the “Search” button, resulting in a list of similar epigenetic enzymes reported in terms of E-values. A significant hit reveals the possibility that the query protein may act with epigenetic regulation in a way similar to the template deposited in HEMD. In addition, the specific catalytic site in the concerned epigenetic enzyme could be validated by alignment to other family proteins. “Modulator search” can be used to design novel epigenetic compounds of known epigenetic enzymes in HEMD. User may paste a SMILES (simplified molecular-input line-entry specification) string [34] or sketch (through Marvin's freely available chemical sketching applet) a potential epigenetic compound into the “Modulator search” window. Submitting the query launches a structure similarity search tool that looks for common features from the query compound that match known epigenetic modulators in HEMD. High-score hits are ranked in a tabular format with hyperlinks to the corresponding full description and in turn to links to the epigenetic enzyme target. The “Modulator search” tool allows users to quickly determine whether their compound of interest acts on the desired epigenetic enzyme target and reveal whether the compound of interest may unexpectedly interact with unintended epigenetic enzyme targets. In addition to these structure similarity searches, the “Modulator search” utility also supports compound searches on the basis of physicochemical properties and chemical formulas. “Text search” provides users a global tool to search throughout HEMD by typing a single term, such as a name, a PDB identifier, or a therapeutics that is related to an epigenetic molecule of interest and the server will return a list of links to relevant entries. Each entry contains a brief introduction of the epigenetic molecule with a hyperlink to its full page.

The “Browse” tools in the database facilitate easy retrieval of information from HEMD through three categories: “Modulator browse”, “Enzyme browse” and “Index browse”. “Modulator browse” is used to visualize all epigenetic modulators with 2D structures and synoptic description in the tab of ‘All’ or three respective categories (“Activator”, “Inhibitor”, and “Regulator”) at the first level, in which each entry links to its second level for exhaustive description of interest. The detailed annotation contains name of the molecule, molecular weight, interactive applets for viewing 2D and 3D molecular structures, >20 drug-like physicochemical properties, experimentally binding affinities, methods, validated epigenetic enzyme targets and therapeutics with hyperlinks to references. This is designed for pharmacists and medicinal chemists who work closely with the quantitative structure–property relationship of epigenetic modulator. “Enzyme browse” allows user to preview the list of names of epigenetic enzymes under class tabs of “DNA”, “Histone”, ‘Chromatin’, and “Non-coding RNA” at the first level (Figure 1) and checking on the selected enzyme in the panel will open a new browser window with a detailed view of the corresponding epigenetic enzyme being displayed, including sequence, structure, native mutation, modification, and disease description, by clicking on the link from the first level. As with most biological databases, all of the proteins illustrated in HEMD are hyperlinked to other online databases or tables like UniProt [25], GenBank [24], Enzyme Nomenclature [23], KEGG [35], NCBI Epigenomics [15], Gene Expression Atlas [36], UCSC Genome Browser [37], or DAnCER [20]. By hyperlinking to these particular databases, HEMD is able to provide considerably more information about epigenetic enzymes in both physiological and pathological conditions. “Index browse” allows the browsing of any epigenetic molecules by their names, which are arranged in alphabetical sequence under each initial letter tab.

In addition to the ‘Browse’ and ‘Search’ options, HEMD also offers epigenetic news, meeting, references, background glossary, and current progress of epigenetic drugs under its “Epigenetic Wiki” menu; HEMD release note and data download under its ‘Download’ menu; statistical information and “Expert” platform for communication of epigenetic information to external experts under its “About” menu; and miscellaneous links to other databases under its “Links” menu. To facilitate the use of HEMD, a series of document including “Quick Start”, “FAQs” and “Tutorial” are provided in the “Help” page.

## Discussion

HEMD is a manually curated database dedicated to epigenetic therapeutics involving enzymes and chemical modulators. It is the first online resource of this kind and the data in HEMD are freely available to all potential users. We harvested verified epigenetic enzymes and modulators from scientific articles. Of the 269 epigenetic enzymes in HEMD, 240 (89.22%) proteins have been associated with diseases and 107 (39.78%) proteins can be targeted for therapeutics by chemical modulators. Remarkably, >45% epigenetic modulators in HEMD are not covered by two important bioactive small molecule databases viz, Drugbank [38] and ChEMBL [39]. Even compared with the largest chemical collection PubChem (http://pubchem.ncbi.nlm.nih.gov), there are still 23% unique compounds in HEMD and a great number of compounds deposited in PubChem have no epigenetic annotations, revealing the potential utilities of HEMD in the epigenetic therapeutics.

Our initial collection mainly focused on epigenetic therapeutics by enzymes and their chemical modulators, which have been widely studied in recent 20 years. More than 200 proteins were found as epigenetic regulators in the area of DNA methylation, histones modifications, chromatin remodeling, and non-coding RNA. The formation and regulation of higher-order chromatin architecture derives from various integrants like DNA methylation, histone modifications, histone variants, and architectural proteins [40], which also could be decomposed into the functions from four types of enzymes curated in HMED. In future, we will continue updating the database every six months and respond to “Expert” request within one week.

HEMD provides users with both chemical and biological tools for information mining on epigenetic molecules. We believe such integrative epigenetic data and tools will not only help scientists to find novel relationships for epigenetic therapeutics, but also provide a starting point for biologists and chemists who have interests in entering the field. HEMD is freely available at http://mdl.shsmu.edu.cn/HEMD/.
